# Old Drugs as New Treatments for Neurodegenerative Diseases

**DOI:** 10.3390/ph11020044

**Published:** 2018-05-11

**Authors:** Fernando Durães, Madalena Pinto, Emília Sousa

**Affiliations:** 1 Laboratory of Organic and Pharmaceutical Chemistry, Department of Chemical Sciences, Faculty of Pharmacy, University of Porto, Rua Jorge Viterbo Ferreira, 228, 4050-313 Porto, Portugal; fduraes5@gmail.com (F.D.); madalena@ff.up.pt (M.P.); 2 CIIMAR, Interdisciplinary Center of Marine and Environmental Research, University of Porto, Terminal de Cruzeiros do Porto de Leixões, Avenida General Norton de Matos P, 4450-208 Matosinhos, Portugal

**Keywords:** neurodegenerative diseases, drug repurposing, Alzheimer’s disease, Parkinson’s disease, Huntington’s disease, multiple sclerosis, amyotrophic lateral sclerosis

## Abstract

Neurodegenerative diseases are increasing in number, given that the general global population is becoming older. They manifest themselves through mechanisms that are not fully understood, in many cases, and impair memory, cognition and movement. Currently, no neurodegenerative disease is curable, and the treatments available only manage the symptoms or halt the progression of the disease. Therefore, there is an urgent need for new treatments for this kind of disease, since the World Health Organization has predicted that neurodegenerative diseases affecting motor function will become the second-most prevalent cause of death in the next 20 years. New therapies can come from three main sources: synthesis, natural products, and existing drugs. This last source is known as drug repurposing, which is the most advantageous, since the drug’s pharmacokinetic and pharmacodynamic profiles are already established, and the investment put into this strategy is not as significant as for the classic development of new drugs. There have been several studies on the potential of old drugs for the most relevant neurodegenerative diseases, including Alzheimer’s disease, Parkinson’s disease, Huntington’s disease, Multiple Sclerosis and Amyotrophic Lateral Sclerosis.

## 1. Introduction

Drug repurposing, also known as drug repositioning or drug reprofiling, is an increasing tendency in drug discovery. This means finding new therapeutic applications for existing drugs and this, alongside synthesis and natural products, is one of the main approaches to finding small molecule leads for new therapeutic applications. Recently, there has been an increase in interest in drug repurposing, especially in new combination therapies, or in diseases with an unmet clinical need, such as orphan and neglected diseases. The advantage is the decreased need for investment in drug discovery and optimization, as well as in safety and pharmacokinetic studies, since the profiles of the repurposed drugs are already established. The repurposing of old drugs can be identified by serendipity, observations of side effects, target searching, or novel insights, and they can act either by the same mechanism of action as their traditional use or by new mechanisms [1,2,3].

One of the most used strategies for drug repositioning is the *in silico* screening of compound libraries in new targets [2]. One remarkable example of drug repurposing is the drug thalidomide. First used as an over-the-counter antiemetic for the treatment of pregnancy-associated morning sickness, it was quickly withdrawn after reports of teratogenicity and dysmyelia. However, in 1998, the Food and Drug Administration (FDA) approved thalidomide for the treatment of cutaneous manifestations of erythema nodosum leprosum. In 2006, thalidomide was approved for the treatment of myeloma, due to its antiangiogenic properties [4]. This example proves how important drug repurposing can be in drug discovery. However, many other examples of old drugs with new uses have been described [5,6].

Neurodegenerative diseases (NDs) are age-dependent disorders, with very different pathophysiologies and a lack of understanding of the causes and mechanisms of these diseases, which leads to a lack of treatment. As the global population is increasingly becoming older, so is the prevalence of these diseases that impair the memory, cognition and movement [7,8]. The need for treatment for NDs is urgent, since the World Health Organization (WHO) predicts that in 20 years, NDs that mainly affect motor functions will overtake cancer to become the second-most prevalent cause of death, after cardiovascular diseases [9,10].

**Figure 1:**
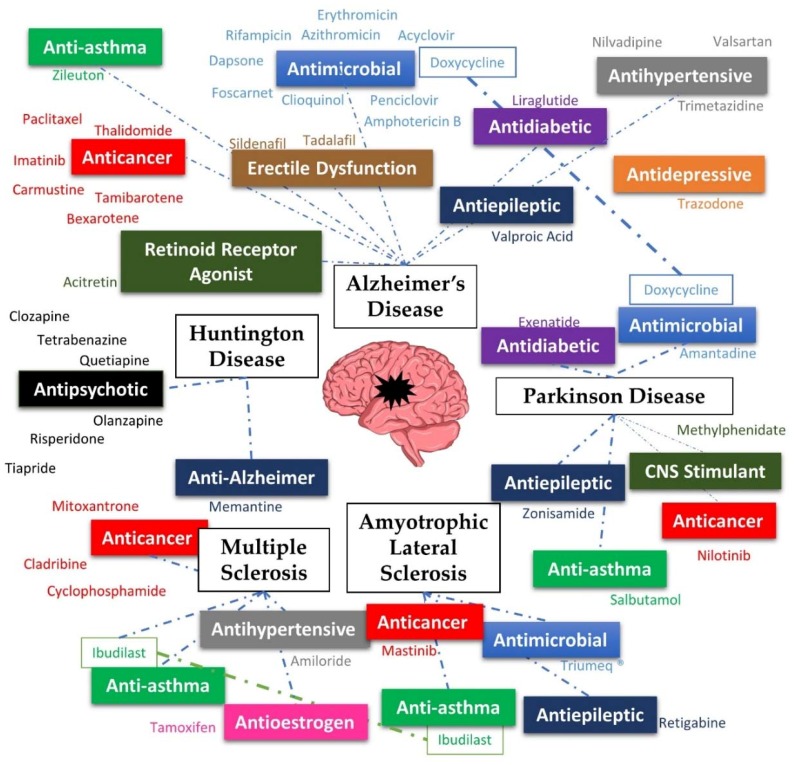
Summary of the diseases and repurposed drugs presented in this review.

With the growing need for treatment for NDs, and the promise that drug repurposing poses, it makes sense that existing drugs are being tested for these diseases. This review comprises drugs that have been repurposed for Alzheimer’s diseases (AD), Parkinson’s disease (PD), Huntington’s disease (HD), multiple sclerosis (MS) and amyotrophic lateral sclerosis (ALS), the most studied neurodegenerative diseases (Figure 1).

Although there have been some reviews concerning the topic of drug repurposing with examples in NDs [11,12,13], there is still no comprehensive review covering this topic for these diseases all together, allowing a broader vision and a better understanding of how these diseases could be related to each other.

## 2. Alzheimer’s Disease

AD is one of the leading NDs, responsible for 80% of dementia cases in elderly people. Its symptoms are the progressive loss of memory, inability to learn, and decline in behaviour and function. The cause for AD is not yet fully understood, but it is believed that it has to do with the deposition of amyloid plaques in the brain, ultimately leading to neuronal and synaptic loss [14,15,16].

**Figure 2:**
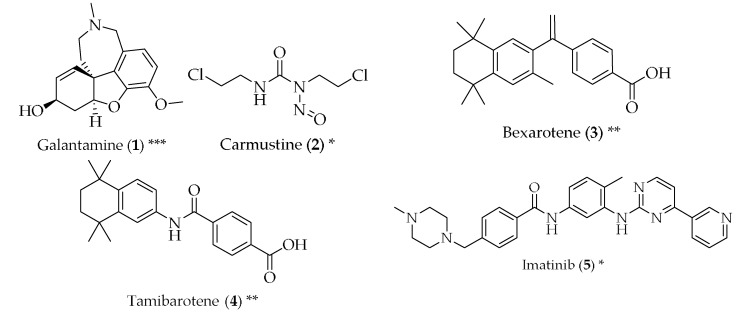

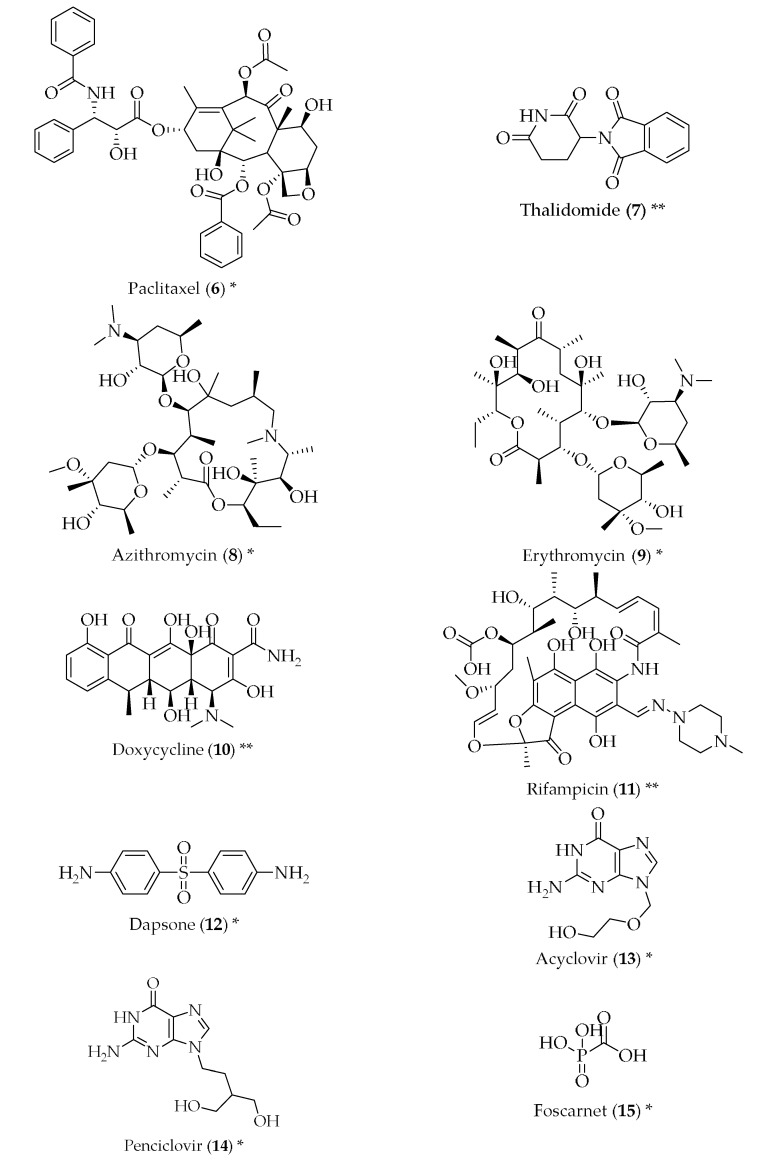

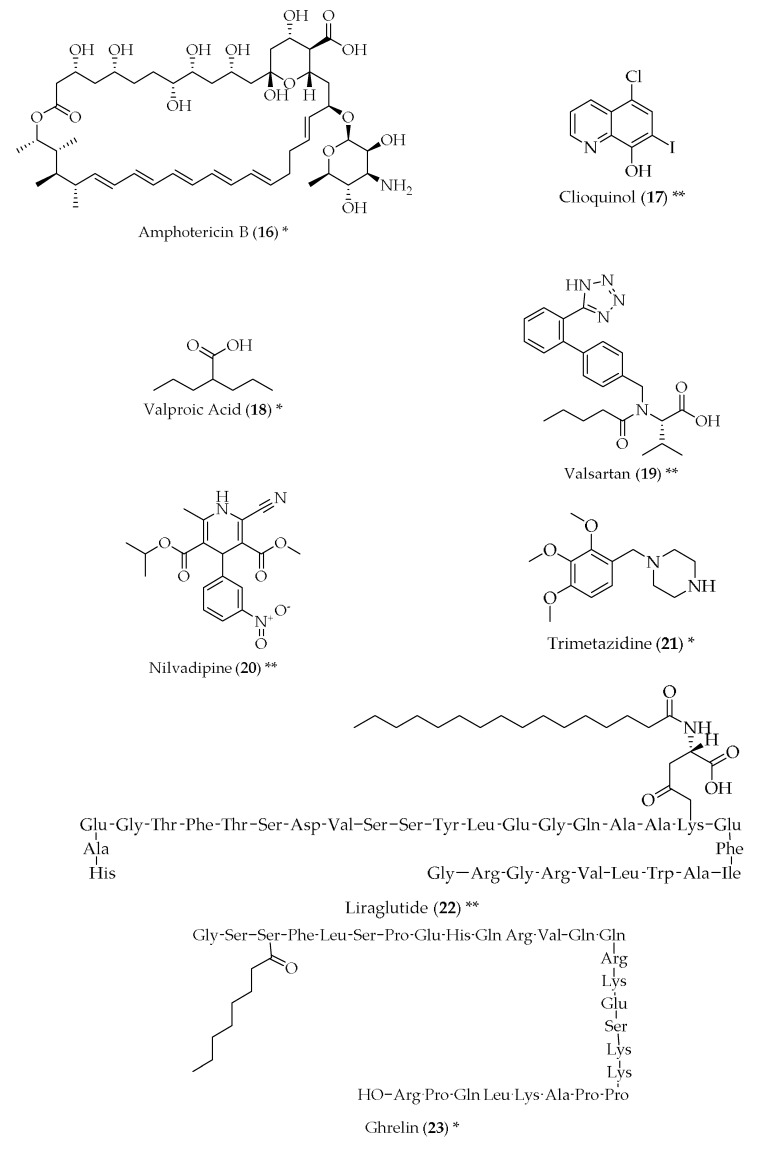

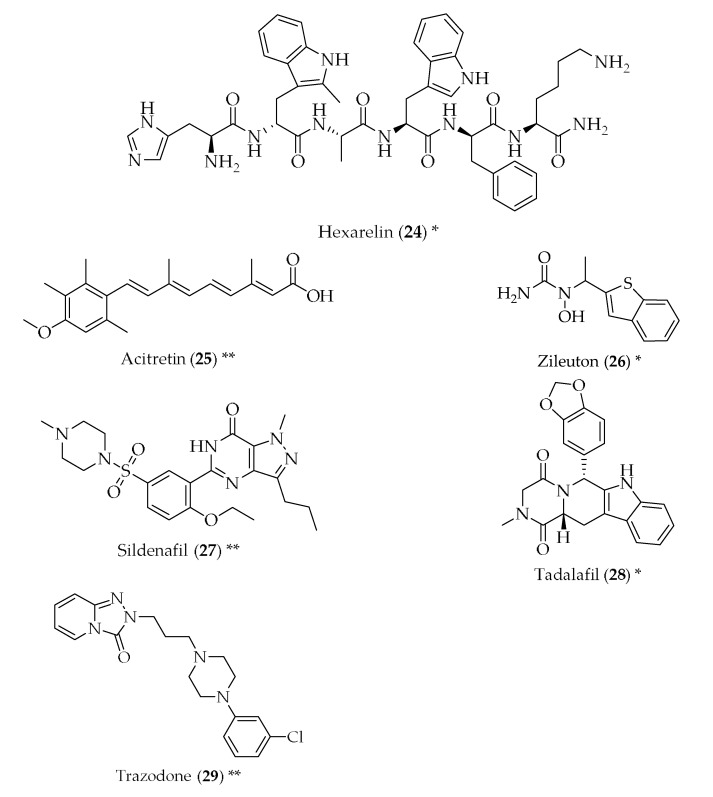
Structure of drugs **1**–**29** (*) Preclinical Studies; (**) Under clinical studies; (***) Clinically approved.

Currently, there is no known cure for AD, and the drugs used within the scope of this disease are mainly to treat the cognitive manifestations or other symptoms and function better when administered at an early stage [14]. Curiously, one of the drugs that is marketed for the treatment of AD, galantamine (**1**, Figure 2), was itself repurposed. In fact, this alkaloid, present in *Galanthus* sp., aroused interest when it was found that it could inhibit muscle acetylcholinesterase, being a good candidate for treating myopathies and peripheral neuropathies, and for the reversal of neuromuscular blockade after anaesthesia, due to the capability of galantamine (**1**) to enhance nerve impulse transmission. As it has a tertiary ammonium base, galantamine (**1**) can easily penetrate the blood-brain barrier and inhibit brain acetylcholinesterase, which makes it particularly interesting [17,18]. In the 1980s, the therapeutic effects of galantamine started being studied for the treatment of AD, and its introduction in the anti-Alzheimer’s armamentarium was made in the year 2000; it still remains one of the most used drugs for delaying the appearance of severe symptoms in patients suffering from AD [18].

Studies have been performed in order to check whether cancer drugs can be repurposed for the treatment of AD. The rationale behind this idea comes from the fact that cancer and neurodegeneration can share signalling pathways, such as mitochondrial dysfunction, oxidative stress, compromised cell metabolism and development of misfolded proteins. It has been described that breast cancer survivors treated with chemotherapy also display a lower risk of developing AD in their old age when compared to a control group [19]. Carmustine (**2**) is a nitrosurea, used as an alkylating agent in brain cancer. It is a small, lipophilic and non-ionized molecule, which makes it capable of penetrating the blood-brain barrier [20]. In cells overexpressing amyloid-β protein precursor, carmustine (**2**) showed a strong reduction in amyloid-β production, at a non-toxic dose [21]. Bexarotene (**3**), a retinoid X receptor antagonist, used to treat cutaneous T-cell lymphomas, has proven to be capable of reversing neurodegeneration, improve cognition and decreasing the levels of amyloid-β in mice overexpressing familial AD mutations [22]. Tamibarotene (**4**), a retinoic acid receptor agonist, approved in Japan for the treatment of acute promyelocytic leukaemia, is able to act on multiple pathways related to the pathophysiology of AD, such as reducing the secretion of proinflammatory cytokines and chemokines by brain cells, improving the behaviour in mice with accelerated senescence and decreasing cortical acetylcholine [23]. Imatinib (**5**), a tyrosine kinase inhibitor approved for the treatment of chronic myelogenous leukaemia and other tumours, has been suggested to be useful for the therapy of AD by two mechanisms: the reduction of amyloid-β and neuroprotection. However, imatinib (**5**) presents the problem of low blood-brain barrier penetration, and it is readily effluxed by P-glycoprotein (P-gp) [24]. Paclitaxel (**6**), an antimitotic agent approved for ovarian and breast cancer, and non-small cell lung cancer, among others, has also been studied as a potential treatment for AD. It is particularly efficient in the treatment of tauopathies, which are defects in the tau protein, which is abundant in cells of the central nervous system and has the function of stabilizing the microtubules. When this protein is phosphorylated, its ability to bind to microtubules is decreased and, therefore, fibrillization is enhanced. Paclitaxel (**6**) reduces this phosphorylation. However, paclitaxel (**6**) has a problem similar to imatinib (**5**): it can be a substrate for P-gp and penetrates the central nervous system poorly [25,26]. Another anticancer drug with potential anti-AD properties is thalidomide (**7**), which has demonstrated inhibition of endothelial cell proliferation, angiogenesis and breakdown of the blood-brain barrier. It was also able to reduce hippocampal neuronal loss through the inhibition of the tumour necrosis factor-α [11,27].

Antimicrobials have also been studied for their potential suitability to treat AD and their symptoms. Both azithromycin (**8**) and erythromycin (**9**), macrolide antibiotics, have shown inhibition of the amyloid precursor protein, resulting in the decrease of cerebral levels of amyloid-β. Tetracyclines have also been proven to reduce the formation of amyloid-β, as well as its resistance to trypsin digestion and an increase in the disassembly of preformed fibrils. They also decreased oxidative stress, suggesting a varied mechanism of action [28]. Doxycycline (**10**) has shown potential in this respect, both alone and in combination with rifampicin (**11**) [29,30,31]. Rifampicin (**11**), most frequently prescribed for *Mycobacterium* infections, has shown effects in the reduction of amyloid-β fibrils, in a dose-dependent manner, probably due to the decreased production and increased clearance of amyloid-β [32]. Dapsone (**12**) is an antibiotic used to treat leprosy, and also received attention in the 1990s, when a decreased incidence of dementia was noticed in leprosy patients that had been treated with dapsone (**12**). Conflicting data about whether or not dapsone (**12**) was capable of decreasing senile plaques [33,34] led to the hypothesis that this event could be a protective factor against amyloid deposition. This hypothesis was further corroborated by studies that showed similar instances of AD in leprosy and tuberculosis patients, in spite of the differences in the percentage of patients that have undergone drug treatments in the two groups [35,36]. The antiviral drugs acyclovir (**13**), penciclovir (**14**) and foscarnet (**15**) have been successful in reducing phosphorylated tau protein and amyloid-β in AD cell models, which can mean they are suitable for the treatment of AD [37]. Amphotericin B (**16**), an antifungal, has been shown to cause a delay in the formation of amyloid-β [38]. However, a more recent study did not reach the same results [39], and the toxicity associated with amphotericin B (**16**) would not make it a suitable candidate for the treatment of AD. Clioquinol (**17**) is an antifungal and antiparasitic drug that has been shown to cause a reduction in the amyloid-β plaques in the brain, with good tolerability in transgenic mice [40].

The antiepileptic drug valproic acid (**18**) has been suggested as a neuroprotective agent for AD, as it has shown reduced formation of amyloid-β plaques and improvement in memory deficits in transgenic mice. The proposed mechanism of action was shown to be complex, but it might be through the inhibition of proinflammatory cytokine production and the enhancement of microglial phagocytosis of amyloid-β [41,42,43,44].

Valsartan (**19**) is an angiotensin receptor blocker, and is used as an antihypertensive. The rationale behind the use of this class of drugs for AD comes from the fact that chronic adverse stress, one of the major environmental causes for the onset and progression of AD, is capable of causing elevations in brain angiotensin II, which act at AT_1_ and AT_2_ receptor subtypes. Furthermore, angiotensin II increases have been suggested to be linked with amyloidogenesis, and the use of angiotensin receptor blockers, blocking AT_1_, appears to be useful in delaying decline in cognitive processing. Apart from this mechanism of action, valsartan (**19**) also inhibits inflammation, vasoconstriction and mitochondrial dysfunction, and promotes the release of acetylcholine [45,46]. Reduced amyloid-β has been reported with in vitro and in vivo treatment of valsartan (**19**), and this evidence suggests a reduction of dementia. Additionally, this drug has good brain penetration, but further studies are required before this drug can be included in the therapy of AD [47,48,49]. Calcium channel blockers are drugs used to treat hypertension and angina. The dihydropyridine calcium channel blockers, such as nilvadipine (**20**), can reduce the production, oligomerization and accumulation of amyloid-β in vitro, improve cell survival and reduce neurotoxicity, while having good blood-brain barrier penetration and increasing brain blood flow through its vasodilatory properties [50,51,52].

Trimetazidine (**21**) is an anti-ischemic drug of the piperazine class. Its mechanism of action is diverse, ranging from increasing nitric oxide production, inhibiting cell apoptosis and being an antioxidant, which increases endothelial function [53]. Apart from being able to pass through the blood-brain barrier, it can reduce the produce of free radicals, due to its antioxidant properties. It can also improve axonal regeneration and effective myelination in healthy and injured nerves [54].

Antidiabetics have also been repurposed for AD, since type 2 diabetes has been identified as a risk factor for AD. Studies have reported a desensitization of insulin signalling in the brains of AD patients. Insulin can also induce neuronal stem cell activation and cell growth and repair, and treatment with insulin has shown neuroprotection and a regulation on the levels of phosphorylated tau protein, as well as an improvement in memory and cognition [55,56,57]. Given this, compounds that influence insulin release can also be useful for AD. Glucagon-like peptide 1 analogues, which promote insulin secretion, may also act in many pathways related to AD, such as the reduction of amyloid-β and the impairment of neuronal function and cell death, as well as tau phosphorylation [58,59]. Liraglutide (**22**) meets these criteria, has established brain penetration and shows physiological effects in the brain, improving learning, and reducing amyloid-β formation and brain inflammation [60,61].

Ghrelin (**23**) is a peptide hormone produced in the gastrointestinal tract that regulates appetite also functioning as a neuropeptide in the central nervous system. It has been demonstrated that ghrelin (**23**), as well as its deacylated form, is capable of inhibiting apoptosis and mitigating the increase of amyloid-β-induced inflammatory interleukins, and manifested neuroprotective effects. It has been hypothesized that when microglia, the first line of immune defence in the brain, interacts with amyloid-β plaques, an inflammatory reaction is initiated. In ND, microglia are overactivated, leading to neuron dysfunction and death. Hexarelin (**24**) and its derivative EP80317 (**25**), synthetic hexapeptides used as a growth hormone stimulant, were able to increase the proliferation of hippocampal progenitor cells in adult rats and protected these cells from necrosis and apoptosis derived from growth-factor withdrawal [62,63,64].

Retinoid receptor activators are used to treat skin conditions, such as acne and psoriasis. Retinoic acid itself is vital for nerve function and repair, and decreases in this compound’s signalling might contribute to AD [65,66,67]. Studies with acitretin (**25**) have shown upregulation of amyloid-clearing enzymes, as well as antioxidant regulation [68].

Zileuton (**26**), an antiasthma drug that acts through the blockage of 5-lipooxygenase, was postulated to have beneficial effects in AD. This comes from the discovery that 5-lipooxygenase is higher in AD patients, making it a promising target within this scope. In fact, studies with zileuton (**26**) in mice showed a reduction in the deposition of amyloid-β [69].

Sildenafil (**27**) and tadalafil (**28**), erectile dysfunction drugs, are inhibitors of phosphodiesterase-5, which is a cGMP controller, which in turn has been linked with AD. cGMP has a detrimental role in controlling learning, memory and neuroplasticity, and may have a positive role in regulating memory impairments resulting from amyloid-β. Sildenafil (**27**) was successful in inhibiting neuroinflammation and was able to lower amyloid-β in aged mice models [70]. Tadalafil (**28**) also displayed cognition enhancement and neuroprotection, while penetrating the blood-brain barrier in concentrations high enough to inhibit phosphodiesterase-5 more effectively than **27** [71].

Recently, trazodone (**29**), an antidepressant, has shown potential in repressing signalling through the PERK/eIF2α-P branch of the unfolded protein response, which plays a detrimental role in controlling protein synthesis in cells and is overactivated in patients with AD. Trazodone (**29**) was successful in reversing eIF2α-P translational attenuation in in vitro and in vivo studies. Furthermore, it displayed neuroprotection, restoration of memory and prevented neurodegeneration [72].

## 3. Parkinson’s Disease

PD is the second-most common ND after AD, and affects populations worldwide. PD is most known for its motor symptoms, which are thought to arise primarily from the loss of dopaminergic neurons within the *substantia nigra*, although other neurotransmitter systems also appear to be affected [73]. It is now known that PD comprises several non-motor related symptoms, such as cognitive impairment, sleep disorders and depression. Even though PD has no cure, the current available treatments are efficacious and keep the disease managed, consisting of dopamine substitution and deep brain stimulation [74].

**Figure 3:**
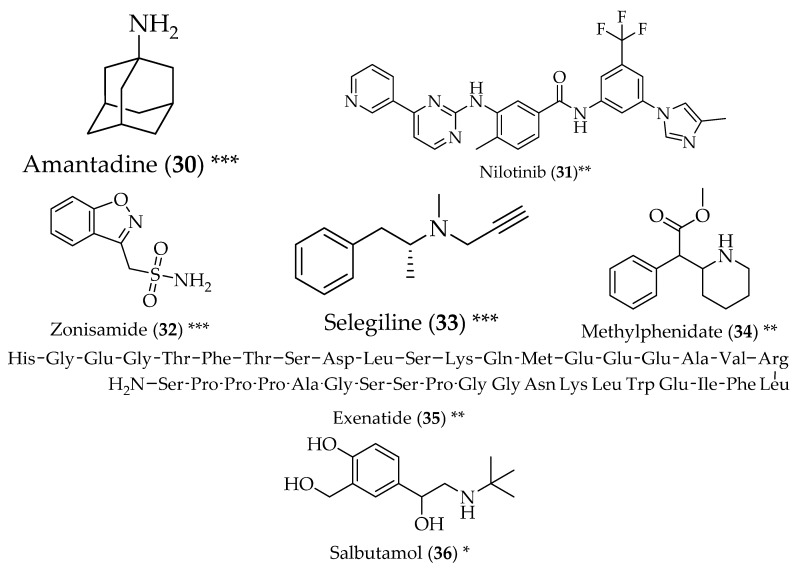
Structure of drugs **30**–**36** (*) Preclinical Studies; (**) Under clinical studies; (***) Clinically approved.

Similar to AD, there is also a drug in the antiparkinsonian arsenal that was itself repurposed: amantadine (**30**, Figure 3). In fact, amantadine (**30**) was first developed to treat influenza, and was only later directed towards the treatment of PD as a weak glutamate receptor antagonist, increasing dopamine and blocking its reuptake [12].

Nilotinib (**31**) is a tyrosine kinase Abl inhibitor that is used for the treatment of chronic myeloid leukaemia. It was observed that Abl is activated in neurodegeneration through the increase in α-synuclein expression and, therefore, its accumulation. Since nilotinib (**31**) inhibits Abl phosphorylation, it increases α-synuclein degradation [75,76].

The antibiotic doxycycline (**10**, Figure 2), previously discussed as a potential anti-AD candidate, has also been studied for its anti-PD effects. In fact, changes in the concentration of doxycycline (**10**) can select between antimicrobial and anti-inflammatory activity. Studies have shown that lower doses than that used for antibiotic purposes do not change bacteria susceptibility, but display anti-inflammatory activity, which is connected to its neuroprotective effects. Other mechanisms that contribute to neuroprotection are doxycycline’s (**10**) antioxidant activity and the ability to remodel early species of α-synuclein oligomers into non-toxic and non-seeding species. Furthermore, doxycycline (**10**) has only been reported to bind to oligomeric species of α-synuclein; the physiological monomeric forms are preserved [77].

Zonisamide (**32**) is a sulphonamide antiepileptic drug, with a mixed mechanism of action, which makes it appropriate for use in different disorders. These mechanisms of action include the blockage of sodium and calcium channels, modulation of the GABA_A_ receptor, inhibition of carbonic anhydrase and inhibition of glutamate release. Studies with rats have shown an increase in dopamine in the striatum when therapeutic doses were used. On the other hand, when higher doses were used, a decrease in intracellular dopamine was observed. Concerning PD, this drug has displayed good activity in both motor and non-motor symptoms, but the mechanism of action is still unclear [78]. Zonisamide (**32**) is also a monoamine oxidase-B inhibitor. This enzyme, mostly present in astrocytes, is responsible for the degradation of dopamine in neural and glial cells, which ultimately leads to the generation of free radicals, which can play a determinant role in the pathogenesis of PD. Its inhibition makes dopamine levels in the synaptic cleft stable and increases the effect of dopamine [79,80]. Another example of a monoamine oxidase-B inhibitor is the antiparkinsonian drug selegiline (**33**), which increases the activation of astrocytes after striatal injury [81,82].

Methylphenidate (**34**) is a central nervous system stimulant that acts through the blockage of the presynaptic dopamine transporter and the noradrenaline transporter, thus inhibiting dopamine and noradrenaline reuptake, in the striatum and the prefrontal cortex. It has been used to treat attention-deficit hyperactivity disorder. Multiple studies with this drug have shown that **34** is effective in reducing gait disorders of PD, as well as non-motor symptoms [83].

Exenatide (**35**) is glucagon-like peptide-1 used for the treatment of type 2 diabetes, like liraglutide, discussed previously. It has been studied as a treatment for PD, and has shown neuroprotection and beneficial neuroplastic change that can delay or prevent disease progression. It can cross the blood-brain barrier, and exerts its neuroprotection through the activation of GLP-1 receptors [84,85,86]. Positive reports in the treatment of AD have also been stated for exenatide (**35**) [87]. Liraglutide (**22**), discussed previously, is currently undergoing phase II clinical trials, with outcomes expected in 2019 (clinicaltrials.gov, ID: NCT02953665).

Antiasthma drugs, namely β2-adrenoreceptor agonists, have been studied for their anti-PD activity. Recent findings have linked the β2-adrenoreceptor with the regulation of the α-synuclein gene *SNCA*. More specifically, β2-adrenoreceptor activation was shown to display neuroprotection. From the drugs tested, three anti-asthmatics were the most promising, with salbutamol (**36**) being the one capable of penetrating the blood-brain barrier and currently approved for treatment. The study undertaken showed that all three drugs were able to reduce the *SNCA*-mRNA and α-synuclein abundance [88].

## 4. Huntington’s Disease

HD is an autosomal dominant disease, and the most common monogenical neurological disease in the developed world. It is characterized by involuntary choreatic movements, behavioural and psychiatric disorders, and dementia. It occurs through a genetic mutation that originates a mutant form of the multifunctional protein huntingtin, which originates toxicity and leads to neuronal death and dysfunction. HD starts to manifest itself in adult life, and the symptoms progress until it leads to death within years. There is no known treatment for this disease, so the only option is the management of the symptoms [89,90].

**Figure 4:**
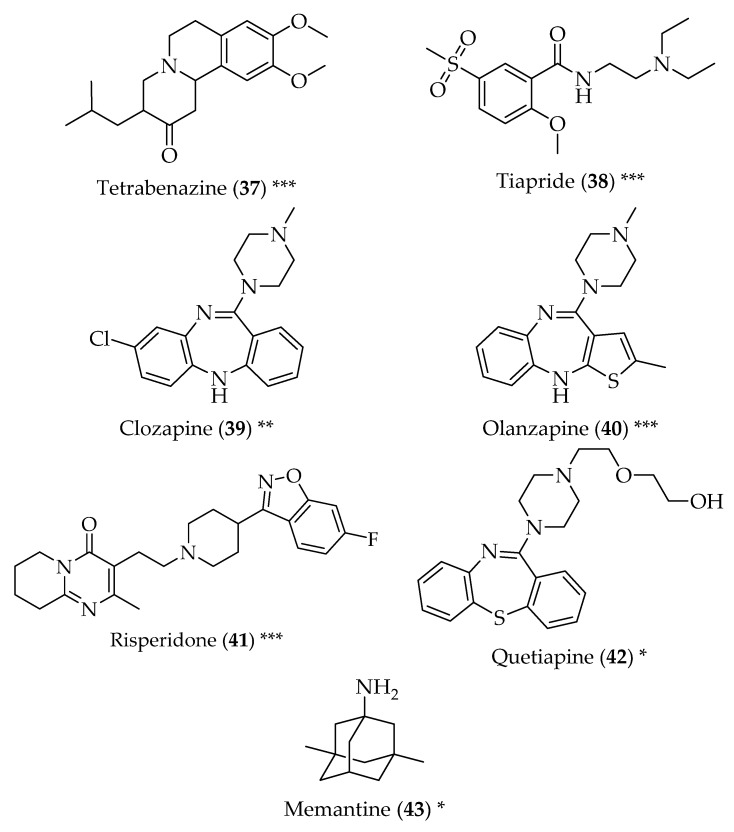
Structure of drugs **37**–**43** (*) Preclinical Studies; (**) Under clinical studies; (***) Clinically approved.

Tetrabenazine (**37**, Figure 4) was first developed as part of research aiming to design simple compounds with reserpine-like antipsychotic activity, acting as a high-affinity, reversible inhibitor of monoamine uptake of presynaptic neurons, and as a weak blocker of the D_2_ dopamine postsynaptic neurons. Antipsychotic studies with this compound were equivocal, and this drug was then repurposed for diseases that manifest themselves by abnormal, involuntary hyperkinetic movements, such as HD. Furthermore, tetrabenazine (**37**) is safer to use in HD than dopamine receptor blocker, since it has never been documented to cause dyskinetic symptoms [91]. Given this, other drugs with dopamine antagonistic activity have been tested for the treatment of HD. This is the case of tiapride (**38**), a D_2_ receptor antagonist, used as an antipsychotic. However, in Europe, selegiline (**33**) is a frequent choice for the treatment of Huntington’s chorea [92]. Clozapine (**39**) is a neuroleptic drug used in the treatment of schizophrenia. It displays a high affinity for the dopamine D_1_ and D_4_ receptors, with low antagonistic activity for the D_2_ dopaminergic receptors. Due to its low incidence of extrapyramidal side effects, it was suggested to be a good symptomatic drug for chorea, although clinical trials showed conflicting results [93]. Olanzapine (**40**), another antipsychotic drug, is also widely prescribed for the treatment of the motor and behavioural symptoms of HD. This drug has high affinity for serotoninergic receptor, but antagonizes dopamine D_2_ receptors. It is also safe and well tolerated, and can be recommended when irritability, sleep dysfunction and weight loss are present, as well as chorea [94,95]. The antipsychotic risperidone (**41**), used in the treatment of schizophrenia and bipolar disorder, acts as a D_2_ receptor antagonist and a serotonin agonist, and therefore can be used for the treatment of HD chorea, as well. It showed beneficial effects on stabilizing motor decline and psychiatric symptoms [96]. The atypical antipsychotic quetiapine (**42**) shows high affinity to serotonin and dopamine receptors. Although not many cases of the use of quetiapine have been described for the treatment of HD symptoms, the few described highlight its usefulness for the treatment of chorea, especially when accompanied by psychiatric symptoms [97].

Memantine (**43**) is an adamantane derivative used for the treatment of AD. It is a non-competitive *N*-methyl-d-aspartate (NMDA) inhibitor. Excessive stimulation of NMDA receptor causes a great influx of calcium into the cell, which ultimately leads to cell death. Therefore, memantine (**43**) can prevent this calcium influx in neuronal cells, and prevent cerebral cell death. Memantine (**43**) was studied for its efficacy in the treatment of HD, and it was noticed that it was able to decrease the vulnerability of neurons to glutamate-mediated excitotoxicity [98,99].

## 5. Multiple Sclerosis

MS is an autoimmune disease of the central nervous system. It is a chronic, inflammatory condition, where the myelin and the axons are destroyed in varying degrees. Its course is unpredictable, and is initially characterized by reversible neurological deficits, which over time become progressive. There is no cure for MS, but there are already therapies approved to reduce the symptoms and progression of the disease [100,101].

**Figure 5:**
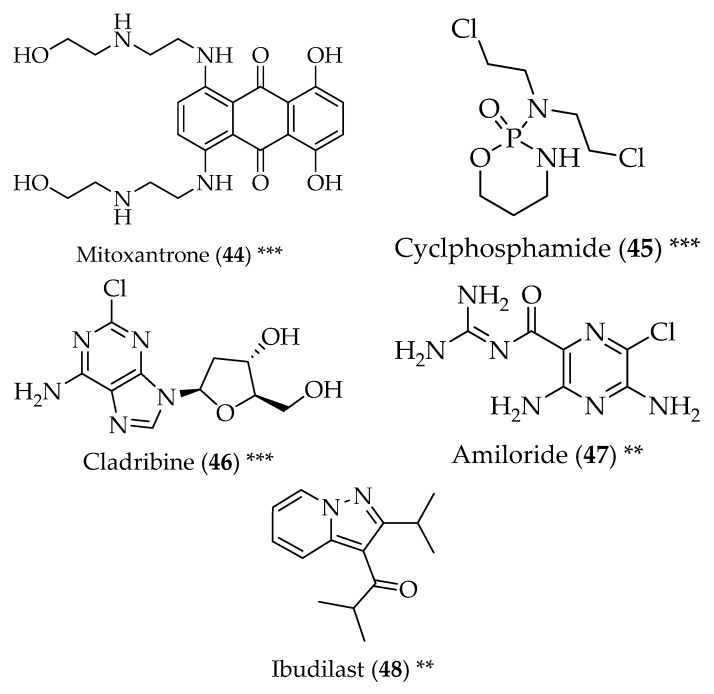
Structure of drugs **44**–**48** (*) Preclinical Studies; (**) Under clinical studies; (***) Clinically approved.

Wide arrays of anticancer drugs have been repurposed for the treatment of MS and its symptoms. This is the case of the synthetic compounds mitoxantrone (**44**, Figure 5), an anthracenedione, established as a wide-spectrum antitumor agent used to treat breast and prostate cancer, acute leukaemia and lymphoma [102]. Mitoxantrone (**44**) has also been approved for the treatment of MS, particularly due to its immunosuppressant properties, associated with erratic responses of the central nervous system T- and B-cells to antigens, myelin damage mediated by macrophages, and axonal injuries. Mitoxantrone (**44**) is capable of inhibiting the activation of T-cells, stopping the proliferation of T- and B-cells, lowering antibody production and deactivating macrophages. Mitoxantrone (**44**) also displayed high tolerability [103]. The alkylating agent cyclophosphamide (**45**) is used to treat a variety of solid tumours, and is approved for the treatment of leukaemia, lymphomas, and breast carcinoma, among others. It is related to nitrogen mustards and binds to DNA, interfering with mitosis and cell replication, targeting mostly rapidly dividing cells. Its use in MS comes from cyclophosphamide (**45**) being able to play an immunosuppressive and immunomodulatory role. Explicitly, it acts in T- and B-cells, supressing cell-mediated and humoral immunity. It has also been shown that cyclophosphamide (**45**) can decrease the secretion of the pro-inflammatory T helper 1 cytokine interferon-γ and interleukin-12, while increasing the secretion of anti-inflammatory cytokines in the brain and blood. It also alters T-lymphocytes into a less inflammatory phenotype. Cyclophosphamide (**45**) can also permeate the blood-brain barrier, having a good bioavailability in the central nervous system, being able to exert its immunomodulation and immunosuppression, thus stabilizing and preventing the progression of the disease [104]. Cladribine (**46**), an antimetabolite, is used to treat hairy cell leukaemia and other hematologic cancers. It is a deoxyadenosine analogue that requires intracellular phosphorylation into a triphosphate in order to become active, which leads to cell death. This drug had been repurposed for the treatment of MS, but was rejected in 2013. Recently, in 2017, cladribine (**46**) was authorized to be marketed as a treatment for this disease by the European Medicines Agency. Its mechanism of action is related to the decrease in circulating B- and T-lymphocytes. Additional mechanisms have been suggested, which is the induction of interferon-α producing myeloid dendritic cells, and the interference with the synaptic effects of interleukin-1β, leading to the conclusion that cladribine (**46**) can also display neuroprotective properties [105,106].

Amiloride (**47**) is a diuretic drug used to treat hypertension and swelling caused by heart failure or liver diseases. It has been studied for its neuroprotective properties in MS. Amiloride can block the neuronal proton-gated acid-sensing ion channel 1 (ASIC1), which is overexpressed in axons and oligodendrocytes in MS lesions, thus exerting its neuroprotective and myeloprotective effects. Furthermore, the fact that amiloride’s protective effect happens downstream of inflammation constitutes an advantage, since it makes it active even on the onset of inflammation [107].

The drug ibudilast (**48**) was approved in some countries for the treatment of bronchial asthma and cerebrovascular disorders. It acts through the inhibition of phosphodiesterases, which are known for their anti-inflammatory effects, but can also inhibit leukotriene and nitric oxide synthesis mechanisms, which are connected to MS. In the brain, ibudilast (**48**) can inhibit the release of the tumour necrosis factor from the microglia and the astrocytes, decreasing neuronal degeneration. Furthermore, it can protect astrocytes from apoptosis and inhibit oligodendrocyte apoptosis and demyelination, hence its usefulness in MS. Studies have shown its safety and tolerability, while reducing the rate of brain atrophy at a high dose [108].

## 6. Amyotrophic Lateral Sclerosis

ALS is a disease characterized by the death of upper and lower motor neurons, which control voluntary muscles. It leads to muscle atrophy, where muscles gradually become weaker, decreasing in size. Other symptoms are muscle stiffness and twitching, and difficulties in breathing, swallowing and speaking. The causes of ALS are of unknown aetiology in most cases, with about 10% being of genetic inheritance [109].

Although some drugs are currently being investigated for their use in the treatment of ALS, only two drugs, riluzole and edaravone, are currently available to delay the progression of the disease, although they cannot revert the symptoms once they have manifested [110,111].

**Figure 6:**
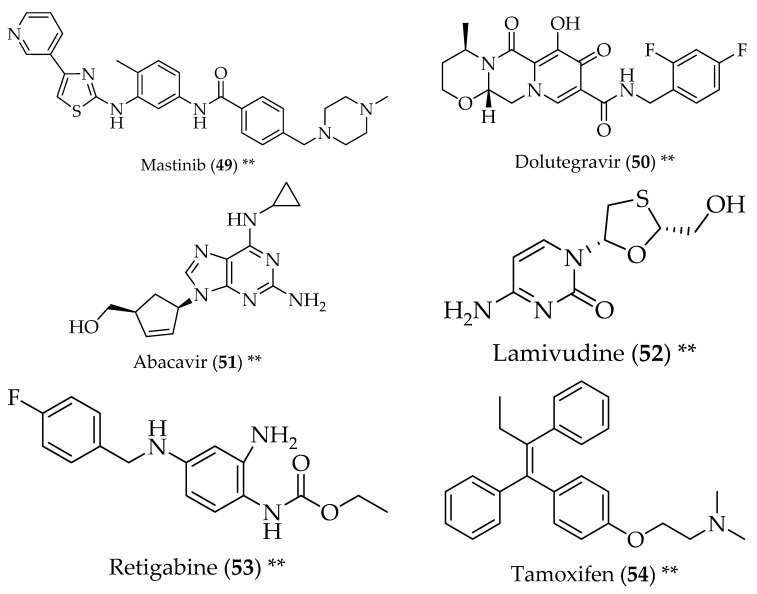
Structure of drugs **49**–**54** (*) Preclinical Studies; (**) Under clinical studies; (***) Clinically approved.

Masitinib (**49**, Figure 6) is a tyrosine kinase inhibitor used to treat cancer in dogs. Its use in ALS resides in the fact that abnormal glial cells that proliferate in ALS might be sensitive to tyrosine kinase inhibitors. It was proven that mastinib (**49**) inhibited glial cell activation in the appropriate rat model and increased survival [112].

Ibudilast (**48**, Figure 5), previously discussed, is also being studied for the treatment of ALS, due to its neuroprotective properties [13].

An antiretroviral, Triumeq^®^, used as an anti-HIV therapy, was studied for the treatment of ALS, based on the fact that ALS patients present serum concentrations of reverse transcriptase comparable to HIV-infected patients, and the expression of a human endogenous retrovirus was noticed in the brain of ALS victims. Taking this into account, anti-HIV drugs can be helpful for ALS. Triumeq ^®^ is a combination of dolutegravir (**50**), an integrase inhibitor, abacavir (**51**) and lamivudine (**52**), antiretrovirals, and has shown safety and tolerability in ALS patients [13].

Retigabine (**53**) is an approved drug for epilepsy, and acts by binding to the voltage-gated potassium channels and increasing the M-current, thus leading to membrane hyperpolarization. Retigabine (**53**) is able to prolong motor neuron survival and decrease excitability, which is advantageous in the treatment of ALS, since it is believed that, in this disease, neurons are hyper-excitable, firing more than normal and ultimately leading to cell death. This drug is still under clinical trial for the treatment of ALS [13].

Tamoxifen (**54**) is an antioestrogen drug, approved for the chemotherapy and chemoprevention of breast cancer. The repurposing of this drug for the treatment of ALS arose serendipitously, after the observation of a neurological improvement in patients and disease stabilization in ALS patients with breast cancer treated with tamoxifen (**54**). Its neuroprotective properties have been described before, and appear to be related to inhibition of protein kinase C, which is overexpressed in the spinal cord of ALS patients. Moreover, tamoxifen (**54**) was found to be able to modulate a proteinopathy present in ALS, through its capacity to be an autophagy modulator [113,114,115].

All the compounds presented in this chapter have been under clinical trial for the treatment of ALS, once this disease has been getting a lot of attention in the last years.

## 7. Failed Repurposing

Although there have been many successful cases of drug repurposing, it is also true that many repositioning attempts have failed. A drug can appear promising at the computational studies level or in in vitro assays, but show no activity in vivo, leading to the abandonment of its study for new activities. This was the case for latrepirdine, an anti-histamine drug approved in Russia for the treatment of rhinitis associated with allergies, repurposing of which was attempted for AD and HD. Even though a mechanism of action had never been clearly established, it had been reported that it was able to modulate the activity of channels and neurotransmitters, blocking amyloid-β toxicity, among other things [116,117]. The fact was that, while phase II studies showed improvement in AD patients over placebo, phase III studies failed to detect significant changes in the disease’s progression [118]. The same happened in phase III studies for HD [119].

Simvastatin and atorvastatin, drugs used to treat hypercholesterolemia, have also been attempted to be repurposed for AD. The theory behind this came from the significant observation that AD and cardiovascular diseases often overlap. Studies had shown that, among other beneficial effects, statins could lower the levels of amyloid-β and increase neuroprotection. However, neither one of them proved to be helpful in the treatment of AD [120,121].

Selective serotonin reuptake inhibitors, used as antidepressants, have also been through studies in order to assess their use in the treatment of AD. Nortriptyline and paroxetine initially demonstrated an improvement in cognitive functions, but further evaluations led to the conclusion that, even after these drugs had treated mood disorders, there was no improvement in cognitive behaviour [122].

The antimicrobial ceftriaxone appeared to be promising in phase II studies for the treatment of ALS, but also failed to show clinical efficacy in phase III studies [123].

Even cladribine was firstly dismissed as a repurposed treatment for MS, before being approved [105]. From these examples it can be inferred that, even though drug repurposing has been shown to be encouraging in the discovery of new treatments for ND, the path that leads to their approval can be problematical, and very often end up in abandonment of the repurposing attempts.

## 8. Conclusions

Through the analysis of the data referred to above, it is clear that drug repurposing can constitute an interesting source of candidates to treat diseases other than those they were originally used for in therapeutics. It also represents an appealing choice, since the costs of repositioning a drug are substantially lower than the costs of designing and optimizing a new drug, as the safety and tolerability have already been established, making clinical studies more cost-effective, requiring smaller samples sizes and reducing the overall cost of the clinical development. With the development of techniques such as high-throughput screening and computational chemistry, old drugs can be tested for new targets easily and quickly, which also constitutes an advantage.

The neurodegenerative diseases presented herein are incurable, and significantly affect the patients’ overall quality of life. The examples of drug repurposing have shown advantages mostly in symptom management and stopping the course of the disease. Unfortunately, a drug has yet to be found that can fully revert the mechanisms of neurodegeneration. However, several drugs presented in this review are already in phase II and phase III clinical trials, meaning that in a few years, the arsenal against neurodegenerative diseases will be much larger than it is today. Other compounds have been through clinical trials but failed to demonstrate efficacy, or displayed high toxicity, as highlighted in Section 7. However, these drugs should not be doomed to abandonment, and they should instead be regarded as a model starting point for the synthesis of new compounds, via molecular modifications and ligand-based design. Pairing this with the continuous progress towards the full understanding of the neuroscience behind psychiatric disorders, repurposing drugs for NDs are likely to be even more promising in the future.

## References

[1] Ashburn T.T., Thor K.B. (2004). Drug repositioning: Identifying and developing new uses for existing drugs. Nat. Rev. Drug Discov..

[2] Doan T.L., Pollastri M., Walters M.A., Georg G.I., Macor J.E. (2011). Chapter 23—The future of drug repositioning: Old drugs, new opportunities. Annual Reports in Medicinal Chemistry.

[3] Fava M. (2018). The promise and challenges of drug repurposing in psychiatry. World Psychiatry.

[4] Richardson P., Hideshima T., Anderson K. (2002). Thalidomide: Emerging role in cancer medicine. Annu. Rev. Med..

[5] Sleire L., Forde H.E., Netland I.A., Leiss L., Skeie B.S., Enger P.O. (2017). Drug repurposing in cancer. Pharmacol. Res..

[6] Baker N.C., Ekins S., Williams A.J., Tropsha A. (2018). A bibliometric review of drug repurposing. Drug Discov. Today.

[7] Agnati L.F., Zoli M., Biagini G., Fuxe K. (1992). Neuronal plasticity and ageing processes in the frame of the ‘red queen theory’. Acta Physiol. Scand..

[8] Agnati L.F., Benfenati F., Solfrini V., Biagini G., Fuxe K., Guidolin D., Carani C., Zini I. (1992). Brain aging and neuronal plasticity. Ann. N. Y. Acad. Sci..

[9] Gitler A.D., Dhillon P., Shorter J. (2017). Neurodegenerative disease: Models, mechanisms, and a new hope. Dis. Models Mech..

[10] Gammon K. (2014). Neurodegenerative disease: Brain windfall. Nature.

[11] Appleby B.S., Nacopoulos D., Milano N., Zhong K., Cummings J.L. (2013). A review: Treatment of alzheimer’s disease discovered in repurposed agents. Dement. Geriatr. Cogn. Disord..

[12] Lee H.-M., Kim Y. (2016). Drug repurposing is a new opportunity for developing drugs against neuropsychiatric disorders. Schizophr. Res. Treat..

[13] Martinez A., Palomo Ruiz M.D., Perez D.I., Gil C. (2017). Drugs in clinical development for the treatment of amyotrophic lateral sclerosis. Expert Opin. Investig. Drugs.

[14] Kumar A., Singh A. (2015). A review on alzheimer’s disease pathophysiology and its management: An update. Pharmacol. Rep..

[15] Scheltens P., Blennow K., Breteler M.M.B., de Strooper B., Frisoni G.B., Salloway S., Van der Flier W.M. (2016). Alzheimer’s disease. Lancet.

[16] Wang J., Gu B.J., Masters C.L., Wang Y.J. (2017). A systemic view of alzheimer disease—Insights from amyloid-beta metabolism beyond the brain. Nat. Rev. Neurol..

[17] Mucke H.A.M. (2015). The case of galantamine: Repurposing and late blooming of a cholinergic drug. Future Sci. OA.

[18] Heinrich M., Cordell G.A. (2010). Chapter 4—Galanthamine from galanthus and other amaryllidaceae—Chemistry and biology based on traditional use. The Alkaloids: Chemistry and Biology.

[19] Monacelli F., Cea M., Borghi R., Odetti P., Nencioni A. (2017). Do cancer drugs counteract neurodegeneration? Repurposing for alzheimer’s disease. J. Alzheimers Dis..

[20] Blakeley J., Grossman S.A., Aminoff M.J., Boller F., Swaab D.F. (2012). Chapter 17—Chemotherapy with cytotoxic and cytostatic agents in brain cancer. Handbook of Clinical Neurology.

[21] Hayes C.D., Dey D., Palavicini J.P., Wang H., Patkar K.A., Minond D., Nefzi A., Lakshmana M.K. (2013). Striking reduction of amyloid plaque burden in an alzheimer’s mouse model after chronic administration of carmustine. BMC Med..

[22] Tousi B. (2015). The emerging role of bexarotene in the treatment of alzheimer’s disease: Current evidence. Neuropsychiatr. Dis. Treat..

[23] Fukasawa H., Nakagomi M., Yamagata N., Katsuki H., Kawahara K., Kitaoka K., Miki T., Shudo K. (2012). Tamibarotene: A candidate retinoid drug for alzheimer’s disease. Biol. Pharm. Bull..

[24] Netzer W.J., Dou F., Cai D., Veach D., Jean S., Li Y., Bornmann W.G., Clarkson B., Xu H., Greengard P. (2003). Gleevec inhibits β-amyloid production but not notch cleavage. Proc. Natl. Acad. Sci. USA.

[25] Brunden K.R., Yao Y., Potuzak J.S., Ferrer N.I., Ballatore C., James M.J., Hogan A.M., Trojanowski J.Q., Smith A.B., Lee V.M. (2011). The characterization of microtubule-stabilizing drugs as possible therapeutic agents for alzheimer’s disease and related tauopathies. Pharmacol. Res..

[26] Zhang B., Maiti A., Shively S., Lakhani F., McDonald-Jones G., Bruce J., Lee E.B., Xie S.X., Joyce S., Li C. (2005). Microtubule-binding drugs offset tau sequestration by stabilizing microtubules and reversing fast axonal transport deficits in a tauopathy model. Proc. Natl. Acad. Sci. USA.

[27] Ryu J.K., McLarnon J.G. (2008). Thalidomide inhibition of perturbed vasculature and glial-derived tumor necrosis factor-alpha in an animal model of inflamed alzheimer’s disease brain. Neurobiol. Dis..

[28] Diomede L., Cassata G., Fiordaliso F., Salio M., Ami D., Natalello A., Doglia S.M., De Luigi A., Salmona M. (2010). Tetracycline and its analogues protect caenorhabditis elegans from beta amyloid-induced toxicity by targeting oligomers. Neurobiol. Dis..

[29] Costa R., Speretta E., Crowther D.C., Cardoso I. (2011). Testing the therapeutic potential of doxycycline in a drosophila melanogaster model of alzheimer disease. J. Biol. Chem..

[30] Loeb M.B., Molloy D.W., Smieja M., Standish T., Goldsmith C.H., Mahony J., Smith S., Borrie M., Decoteau E., Davidson W. (2004). A randomized, controlled trial of doxycycline and rifampin for patients with alzheimer’s disease. J. Am. Geriatr. Soc..

[31] Molloy D.W., Standish T.I., Zhou Q., Guyatt G. (2013). A multicenter, blinded, randomized, factorial controlled trial of doxycycline and rifampin for treatment of alzheimer’s disease: The darad trial. Int. J. Geriatr. Psychiatry.

[32] Tomiyama T., Shoji A., Kataoka K., Suwa Y., Asano S., Kaneko H., Endo N. (1996). Inhibition of amyloid beta protein aggregation and neurotoxicity by rifampicin. Its possible function as a hydroxyl radical scavenger. J. Biol. Chem..

[33] Kimura T., Goto M. (1993). Existence of senile plaques in the brains of elderly leprosy patients. Lancet.

[34] Chui D.H., Tabira T., Izumi S., Koya G., Ogata J. (1994). Decreased beta-amyloid and increased abnormal tau deposition in the brain of aged patients with leprosy. Am. J. Pathol..

[35] Goto M., Kimura T., Hagio S., Ueda K., Kitajima S., Tokunaga H., Sato E. (1995). Neuropathological analysis of dementia in a japanese leprosarium. Dementia.

[36] Endoh M., Kunishita T., Tabira T. (1999). No effect of anti-leprosy drugs in the prevention of alzheimer’s disease and beta-amyloid neurotoxicity. J. Neurol. Sci..

[37] Wozniak M.A., Itzhaki R.F. (2010). Antiviral agents in alzheimer’s disease: Hope for the future?. Ther. Adv. Neurol. Disord..

[38] Hartsel S.C., Weiland T.R. (2003). Amphotericin b binds to amyloid fibrils and delays their formation: A therapeutic mechanism?. Biochemistry.

[39] Smith N.W., Annunziata O., Dzyuba S.V. (2009). Amphotericin b interactions with soluble oligomers of amyloid abeta1-42 peptide. Bioorg. Med. Chem..

[40] Grossi C., Francese S., Casini A., Rosi M.C., Luccarini I., Fiorentini A., Gabbiani C., Messori L., Moneti G., Casamenti F. (2009). Clioquinol decreases amyloid-beta burden and reduces working memory impairment in a transgenic mouse model of alzheimer’s disease. J. Alzheimers Dis..

[41] Mark R.J., Ashford J.W., Goodman Y., Mattson M.P. (1995). Anticonvulsants attenuate amyloid beta-peptide neurotoxicity, Ca^2+^ deregulation, and cytoskeletal pathology. Neurobiol. Aging.

[42] Smith A.M., Gibbons H.M., Dragunow M. (2010). Valproic acid enhances microglial phagocytosis of amyloid-beta(1–42). Neuroscience.

[43] Qing H., He G., Ly P.T., Fox C.J., Staufenbiel M., Cai F., Zhang Z., Wei S., Sun X., Chen C.H. (2008). Valproic acid inhibits abeta production, neuritic plaque formation, and behavioral deficits in alzheimer’s disease mouse models. J. Exp. Med..

[44] Tariot P.N., Schneider L.S., Cummings J., Thomas R.G., Raman R., Jakimovich L.J., Loy R., Bartocci B., Fleisher A., Ismail M.S. (2011). Chronic divalproex sodium to attenuate agitation and clinical progression of alzheimer disease. Arch. Gen. Psychiatry.

[45] Culman J., Blume A., Gohlke P., Unger T. (2002). The renin-angiotensin system in the brain: Possible therapeutic implications for at(1)-receptor blockers. J. Hum. Hypertens..

[46] Wright J.W., Harding J.W. (2011). Brain renin-angiotensin—A new look at an old system. Prog. Neurobiol..

[47] Wang J., Ho L., Chen L., Zhao Z., Zhao W., Qian X., Humala N., Seror I., Bartholomew S., Rosendorff C. (2007). Valsartan lowers brain β-amyloid protein levels and improves spatial learning in a mouse model of alzheimer disease. J. Clin. Investig..

[48] Li N.C., Lee A., Whitmer R.A., Kivipelto M., Lawler E., Kazis L.E., Wolozin B. (2010). Use of angiotensin receptor blockers and risk of dementia in a predominantly male population: Prospective cohort analysis. BMJ.

[49] Danielyan L., Klein R., Hanson L.R., Buadze M., Schwab M., Gleiter C.H., Frey W.H. (2010). Protective effects of intranasal losartan in the app/ps1 transgenic mouse model of alzheimer disease. Rejuvenation Res..

[50] Hanyu H., Hirao K., Shimizu S., Iwamoto T., Koizumi K., Abe K. (2007). Favourable effects of nilvadipine on cognitive function and regional cerebral blood flow on spect in hypertensive patients with mild cognitive impairment. Nucl. Med. Commun..

[51] Zhao W., Wang J., Ho L., Ono K., Teplow D.B., Pasinetti G.M. (2009). Identification of antihypertensive drugs which inhibit amyloid-beta protein oligomerization. J. Alzheimers Dis..

[52] Bachmeier C., Beaulieu-Abdelahad D., Mullan M., Paris D. (2011). Selective dihydropyiridine compounds facilitate the clearance of beta-amyloid across the blood-brain barrier. Eur. J. Pharmacol..

[53] Zou H., Zhu X.X., Ding Y.H., Jin Q.Y., Qian L.Y., Huang D.S., Cen X.J. (2017). Trimetazidine in conditions other than coronary disease, old drug, new tricks?. Int. J. Cardiol..

[54] Hassanzadeh G., Hosseini A., Pasbakhsh P., Akbari M., Ghaffarpour M., Takzare N., Zahmatkesh M. (2015). Trimetazidine prevents oxidative changes induced in a rat model of sporadic type of alzheimer’s disease. Acta Med. Iran..

[55] Carro E., Torres-Aleman I. (2004). The role of insulin and insulin-like growth factor i in the molecular and cellular mechanisms underlying the pathology of alzheimer’s disease. Eur. J. Pharmacol..

[56] Watson G.S., Craft S. (2004). Modulation of memory by insulin and glucose: Neuropsychological observations in alzheimer’s disease. Eur. J. Pharmacol..

[57] Zhao W.Q., Chen H., Quon M.J., Alkon D.L. (2004). Insulin and the insulin receptor in experimental models of learning and memory. Eur. J. Pharmacol..

[58] Perry T., Lahiri D.K., Sambamurti K., Chen D., Mattson M.P., Egan J.M., Greig N.H. (2003). Glucagon-like peptide-1 decreases endogenous amyloid-beta peptide (abeta) levels and protects hippocampal neurons from death induced by abeta and iron. J. Neurosci. Res..

[59] Perry T., Lahiri D.K., Chen D., Zhou J., Shaw K.T., Egan J.M., Greig N.H. (2002). A novel neurotrophic property of glucagon-like peptide 1: A promoter of nerve growth factor-mediated differentiation in pc12 cells. J. Pharmacol. Exp. Ther..

[60] McClean P.L., Parthsarathy V., Faivre E., Holscher C. (2011). The diabetes drug liraglutide prevents degenerative processes in a mouse model of alzheimer’s disease. J. Neurosci..

[61] Xiong H., Zheng C., Wang J., Song J., Zhao G., Shen H., Deng Y. (2013). The neuroprotection of liraglutide on alzheimer-like learning and memory impairment by modulating the hyperphosphorylation of tau and neurofilament proteins and insulin signaling pathways in mice. J. Alzheimers Dis..

[62] Wagner J., Vulinovic F., Grunewald A., Unger M.M., Moller J.C., Klein C., Michel P.P., Ries V., Oertel W.H., Alvarez-Fischer D. (2017). Acylated and unacylated ghrelin confer neuroprotection to mesencephalic neurons. Neuroscience.

[63] Lucchi C., Curia G., Vinet J., Gualtieri F., Bresciani E., Locatelli V., Torsello A., Biagini G. (2013). Protective but not anticonvulsant effects of ghrelin and jmv-1843 in the pilocarpine model of status epilepticus. PLoS ONE.

[64] Bulgarelli I., Tamiazzo L., Bresciani E., Rapetti D., Caporali S., Lattuada D., Locatelli V., Torsello A. (2009). Desacyl-ghrelin and synthetic gh-secretagogues modulate the production of inflammatory cytokines in mouse microglia cells stimulated by beta-amyloid fibrils. J. Neurosci. Res..

[65] Ding Y., Qiao A., Wang Z., Goodwin J.S., Lee E.-S., Block M.L., Allsbrook M., McDonald M.P., Fan G.-H. (2008). Retinoic acid attenuates β-amyloid deposition and rescues memory deficits in an alzheimer’s disease transgenic mouse model. J. Neurosci..

[66] Jarvis C.I., Goncalves M.B., Clarke E., Dogruel M., Kalindjian S.B., Thomas S.A., Maden M., Corcoran J.P. (2010). Retinoic acid receptor-alpha signalling antagonizes both intracellular and extracellular amyloid-beta production and prevents neuronal cell death caused by amyloid-beta. Eur. J. Neurosci..

[67] Shudo K., Fukasawa H., Nakagomi M., Yamagata N. (2009). Towards retinoid therapy for alzheimer’s disease. Curr. Alzheimer Res..

[68] Tippmann F., Hundt J., Schneider A., Endres K., Fahrenholz F. (2009). Up-regulation of the alpha-secretase adam10 by retinoic acid receptors and acitretin. FASEB J..

[69] Di Meco A., Lauretti E., Vagnozzi A.N., Praticò D. (2014). Zileuton restores memory impairments and reverses amyloid and tau pathology in aged ad mice. Neurobiol. Aging.

[70] Zhang J., Guo J., Zhao X., Chen Z., Wang G., Liu A., Wang Q., Zhou W., Xu Y., Wang C. (2013). Phosphodiesterase-5 inhibitor sildenafil prevents neuroinflammation, lowers beta-amyloid levels and improves cognitive performance in app/ps1 transgenic mice. Behav. Brain Res..

[71] Garcia-Barroso C., Ricobaraza A., Pascual-Lucas M., Unceta N., Rico A.J., Goicolea M.A., Salles J., Lanciego J.L., Oyarzabal J., Franco R. (2013). Tadalafil crosses the blood-brain barrier and reverses cognitive dysfunction in a mouse model of ad. Neuropharmacology.

[72] Halliday M., Radford H., Zents K.A.M., Molloy C., Moreno J.A., Verity N.C., Smith E., Ortori C.A., Barrett D.A., Bushell M. (2017). Repurposed drugs targeting eif2α-p-mediated translational repression prevent neurodegeneration in mice. Brain.

[73] Antony P.M., Diederich N.J., Kruger R., Balling R. (2013). The hallmarks of parkinson’s disease. FEBS J..

[74] Poewe W., Seppi K., Tanner C.M., Halliday G.M., Brundin P., Volkmann J., Schrag A.-E., Lang A.E. (2017). Parkinson disease. Nat. Rev. Dis. Prim..

[75] Pagan F., Hebron M., Valadez E.H., Torres-Yaghi Y., Huang X., Mills R.R., Wilmarth B.M., Howard H., Dunn C., Carlson A. (2016). Nilotinib effects in parkinson’s disease and dementia with lewy bodies. J. Parkinsons Dis..

[76] Hebron M.L., Lonskaya I., Moussa C.E.H. (2013). Nilotinib reverses loss of dopamine neurons and improves motor behavior via autophagic degradation of α-synuclein in parkinson’s disease models. Hum. Mol. Genet..

[77] González-Lizárraga F., Socías S.B., Ávila C.L., Torres-Bugeau C.M., Barbosa L.R.S., Binolfi A., Sepúlveda-Díaz J.E., Del-Bel E., Fernandez C.O., Papy-Garcia D. (2017). Repurposing doxycycline for synucleinopathies: Remodelling of α-synuclein oligomers towards non-toxic parallel beta-sheet structured species. Sci. Rep..

[78] Bermejo P.E., Anciones B. (2009). A review of the use of zonisamide in parkinson’s disease. Ther. Adv. Neurol. Disord..

[79] Fox S.H., Katzenschlager R., Lim S.Y., Barton B., de Bie R.M.A., Seppi K., Coelho M., Sampaio C. (2018). International parkinson and movement disorder society evidence-based medicine review: Update on treatments for the motor symptoms of parkinson’s disease. Mov. Disord..

[80] Riederer P., Muller T. (2018). Monoamine oxidase-b inhibitors in the treatment of parkinson’s disease: Clinical-pharmacological aspects. J. Neural. Transm..

[81] Biagini G., Zoli M., Fuxe K., Agnati L.F. (1993). L-deprenyl increases gfap immunoreactivity selectively in activated astrocytes in rat brain. Neuroreport.

[82] Biagini G., Frasoldati A., Fuxe K., Agnati L.F. (1994). The concept of astrocyte-kinetic drug in the treatment of neurodegenerative diseases: Evidence for l-deprenyl-induced activation of reactive astrocytes. Neurochem. Int..

[83] Devos D., Moreau C., Delval A., Dujardin K., Defebvre L., Bordet R. (2013). Methylphenidate: A treatment for parkinson’s disease?. CNS Drugs.

[84] Jankovic J. (2017). Exenatide—A drug for diabetes and parkinson disease?. Nat. Rev. Neurol..

[85] Athauda D., Wyse R., Brundin P., Foltynie T. (2017). Is exenatide a treatment for parkinson’s disease?. J. Parkinsons Dis..

[86] Aviles-Olmos I., Dickson J., Kefalopoulou Z., Djamshidian A., Ell P., Soderlund T., Whitton P., Wyse R., Isaacs T., Lees A. (2013). Exenatide and the treatment of patients with parkinson’s disease. J. Clin. Investig..

[87] Bomba M., Ciavardelli D., Silvestri E., Canzoniero L.M., Lattanzio R., Chiappini P., Piantelli M., Di Ilio C., Consoli A., Sensi S.L. (2013). Exenatide promotes cognitive enhancement and positive brain metabolic changes in ps1-ki mice but has no effects in 3xtg-ad animals. Cell Death Dis..

[88] Mittal S., Bjørnevik K., Im D.S., Flierl A., Dong X., Locascio J.J., Abo K.M., Long E., Jin M., Xu B. (2017). Β2-adrenoreceptor is a regulator of the α-synuclein gene driving risk of parkinson’s disease. Science.

[89] Bates G.P., Dorsey R., Gusella J.F., Hayden M.R., Kay C., Leavitt B.R., Nance M., Ross C.A., Scahill R.I., Wetzel R. (2015). Huntington disease. Nat. Rev. Dis. Prim..

[90] Roos R.A. (2010). Huntington’s disease: A clinical review. Orphanet J. Rare Dis..

[91] Paleacu D. (2007). Tetrabenazine in the treatment of huntington’s disease. Neuropsychiatr. Dis. Treat..

[92] Roos R.A., Buruma O.J., Bruyn G.W., Kemp B., van der Velde E.A. (1982). Tiapride in the treatment of huntington’s chorea. Acta Neurol. Scand..

[93] Bonuccelli U., Ceravolo R., Maremmani C., Nuti A., Rossi G., Muratorio A. (1994). Clozapine in huntington’s chorea. Neurology.

[94] Paleacu D., Anca M., Giladi N. (2002). Olanzapine in huntington’s disease. Acta Neurol. Scand..

[95] Coppen E.M., Roos R.A.C. (2017). Current pharmacological approaches to reduce chorea in huntington’s disease. Drugs.

[96] Duff K., Beglinger L.J., O’Rourke M.E., Nopoulos P., Paulson H.L., Paulson J.S. (2008). Risperidone and the treatment of psychiatric, motor, and cognitive symptoms in huntington’s disease. Ann. Clin. Psychiatry.

[97] Alpay M., Koroshetz W.J. (2006). Quetiapine in the treatment of behavioral disturbances in patients with huntington’s disease. Psychosomatics.

[98] Beister A., Kraus P., Kuhn W., Dose M., Weindl A., Gerlach M. (2004). The n-methyl-d-aspartate antagonist memantine retards progression of huntington’s disease. J. Neural Transm. Suppl..

[99] Anitha M., Nandhu M.S., Anju T.R., Jes P., Paulose C.S. (2011). Targeting glutamate mediated excitotoxicity in huntington’s disease: Neural progenitors and partial glutamate antagonist--memantine. Med. Hypotheses.

[100] Goldenberg M.M. (2012). Multiple sclerosis review. P&T.

[101] Trapp B.D., Nave K.A. (2008). Multiple sclerosis: An immune or neurodegenerative disorder?. Annu. Rev. Neurosci..

[102] Hrynchak I., Sousa E., Pinto M., Costa V.M. (2017). The importance of drug metabolites synthesis: The case-study of cardiotoxic anticancer drugs. Drug Metab. Rev..

[103] Hartung H.-P., Gonsette R., Konig N., Kwiecinski H., Guseo A., Morrissey S.P., Krapf H., Zwingers T. (2002). Mitoxantrone in progressive multiple sclerosis: A placebo-controlled, double-blind, randomised, multicentre trial. Lancet.

[104] Awad A., Stuve O. (2009). Cyclophosphamide in multiple sclerosis: Scientific rationale, history and novel treatment paradigms. Ther. Adv. Neurol. Disord..

[105] Leist T.P., Weissert R. (2011). Cladribine: Mode of action and implications for treatment of multiple sclerosis. Clin. Neuropharmacol..

[106] Holmoy T., Torkildsen O., Myhr K.M. (2017). An update on cladribine for relapsing-remitting multiple sclerosis. Expert Opin. Pharmacother..

[107] Arun T., Tomassini V., Sbardella E., de Ruiter M.B., Matthews L., Leite M.I., Gelineau-Morel R., Cavey A., Vergo S., Craner M. (2013). Targeting asic1 in primary progressive multiple sclerosis: Evidence of neuroprotection with amiloride. Brain.

[108] Barkhof F., Hulst H.E., Drulovic J., Uitdehaag B.M., Matsuda K., Landin R. (2010). Ibudilast in relapsing-remitting multiple sclerosis: A neuroprotectant?. Neurology.

[109] Rowland L.P., Shneider N.A. (2001). Amyotrophic lateral sclerosis. N. Engl. J. Med..

[110] Zoccolella S., Beghi E., Palagano G., Fraddosio A., Guerra V., Samarelli V., Lepore V., Simone I.L., Lamberti P., Serlenga L. (2007). Riluzole and amyotrophic lateral sclerosis survival: A population-based study in southern italy. Eur. J. Neurol..

[111] Sawada H. (2017). Clinical efficacy of edaravone for the treatment of amyotrophic lateral sclerosis. Expert Opin. Pharmacother..

[112] Trias E., Ibarburu S., Barreto-Núñez R., Babdor J., Maciel T.T., Guillo M., Gros L., Dubreuil P., Díaz-Amarilla P., Cassina P. (2016). Post-paralysis tyrosine kinase inhibition with masitinib abrogates neuroinflammation and slows disease progression in inherited amyotrophic lateral sclerosis. J. Neuroinflamm..

[113] Goodman A. (2005). Tamoxifen, a cancer therapy, explored for als. Neurol. Today.

[114] Hu J.H., Zhang H., Wagey R., Krieger C., Pelech S.L. (2003). Protein kinase and protein phosphatase expression in amyotrophic lateral sclerosis spinal cord. J. Neurochem..

[115] Wang I.F., Guo B.S., Liu Y.C., Wu C.C., Yang C.H., Tsai K.J., Shen C.K. (2012). Autophagy activators rescue and alleviate pathogenesis of a mouse model with proteinopathies of the tar DNA-binding protein 43. Proc. Natl. Acad. Sci. USA.

[116] Bezprozvanny I. (2010). The rise and fall of dimebon. Drug News Perspect..

[117] Bharadwaj P.R., Bates K.A., Porter T., Teimouri E., Perry G., Steele J.W., Gandy S., Groth D., Martins R.N., Verdile G. (2013). Latrepirdine: Molecular mechanisms underlying potential therapeutic roles in alzheimer’s and other neurodegenerative diseases. Transl. Psychiatry.

[118] Doody R.S., Gavrilova S.I., Sano M., Thomas R.G., Aisen P.S., Bachurin S.O., Seely L., Hung D. (2008). Effect of dimebon on cognition, activities of daily living, behaviour, and global function in patients with mild-to-moderate alzheimer’s disease: A randomised, double-blind, placebo-controlled study. Lancet.

[119] Cano-Cuenca N., Solis-Garcia del Pozo J.E., Jordan J. (2014). Evidence for the efficacy of latrepirdine (dimebon) treatment for improvement of cognitive function: A meta-analysis. J. Alzheimers Dis..

[120] Sano M., Bell K.L., Galasko D., Galvin J.E., Thomas R.G., van Dyck C.H., Aisen P.S. (2011). A randomized, double-blind, placebo-controlled trial of simvastatin to treat alzheimer disease. Neurology.

[121] Sparks D.L., Sabbagh M.N., Connor D.J., Lopez J., Launer L.J., Petanceska S., Browne P., Wassar D., Johnson-Traver S., Lochhead J. (2005). Atorvastatin therapy lowers circulating cholesterol but not free radical activity in advance of identifiable clinical benefit in the treatment of mild-to-moderate ad. Curr. Alzheimer Res..

[122] Nebes R.D., Pollock B.G., Houck P.R., Butters M.A., Mulsant B.H., Zmuda M.D., Reynolds C.F. (2003). Persistence of cognitive impairment in geriatric patients following antidepressant treatment: A randomized, double-blind clinical trial with nortriptyline and paroxetine. J. Psychiatr. Res..

[123] Cudkowicz M.E., Titus S., Kearney M., Yu H., Sherman A., Schoenfeld D., Hayden D., Shui A., Brooks B., Conwit R. (2014). Efficacy and safety of ceftriaxone for amyotrophic lateral sclerosis: Results of a multi-stage, randomised, double-blind, placebo-controlled, phase 3 study. Lancet.

